# High-fat diet accelerates extreme obesity with hyperphagia in female heterozygous *Mecp2*-null mice

**DOI:** 10.1371/journal.pone.0210184

**Published:** 2019-01-04

**Authors:** Shota Fukuhara, Hisakazu Nakajima, Satoru Sugimoto, Kazuki Kodo, Keiichi Shigehara, Hidechika Morimoto, Yusuke Tsuma, Masaharu Moroto, Jun Mori, Kitaro Kosaka, Masafumi Morimoto, Hajime Hosoi

**Affiliations:** 1 Department of Pediatrics, Graduate School of Medical Science, Kyoto Prefectural University of Medicine, Kyoto City, Japan; 2 Department of Pediatrics, North Medical Center, Kyoto, Prefectural University of Medicine, Yosa-gun, Japan; University of Melbourne, AUSTRALIA

## Abstract

Rett syndrome (RTT) is an X-linked neurodevelopmental disorder caused by mutation of the methyl-CpG-binding protein 2 (*MECP2*) gene. Although RTT has been associated with obesity, the underlying mechanism has not yet been elucidated. In this study, female heterozygous *Mecp2*-null mice (*Mecp2*^+/-^ mice), a model of RTT, were fed a normal chow diet or high-fat diet (HFD), and the changes in molecular signaling pathways were investigated. Specifically, we examined the expression of genes related to the hypothalamus and dopamine reward circuitry, which represent a central network of feeding behavior control. In particular, dopamine reward circuitry has been shown to regulate hedonic feeding behavior, and its disruption is associated with HFD-related changes in palatability. The *Mecp2*^+/-^ mice that were fed the normal chow showed normal body weight and food consumption, whereas those fed the HFD showed extreme obesity with hyperphagia, an increase of body fat mass, glucose intolerance, and insulin resistance compared with wild-type mice fed the HFD (WT-HFD mice). The main cause of obesity in *Mecp2*^+/-^-HFD mice was a remarkable increase in calorie intake, with no difference in oxygen consumption or locomotor activity. Agouti-related peptide mRNA and protein levels were increased, whereas proopiomelanocortin mRNA and protein levels were reduced in *Mecp2*^*+/-*^-HFD mice with hyperleptinemia, which play an essential role in appetite and satiety in the hypothalamus. The conditioned place preference test revealed that *Mecp2*^*+/-*^ mice preferred the HFD. Tyrosine hydroxylase and dopamine transporter mRNA levels in the ventral tegmental area, and dopamine receptor and dopamine- and cAMP-regulated phosphoprotein mRNA levels in the nucleus accumbens were significantly lower in *Mecp2*^*+/-*^-HFD mice than those of WT-HFD mice. Thus, HFD feeding induced dysregulation of food intake in the hypothalamus and dopamine reward circuitry, and accelerated the development of extreme obesity associated with addiction-like eating behavior in *Mecp2*^*+/-*^ mice.

## Introduction

Rett syndrome (RTT) is a neurodevelopmental disorder resulting from mutation of the methyl-CpG-binding protein 2 (*MECP2*) gene on the X chromosome. RTT is characterized by developmental regression, autistic behavior, stereotypical hand movements, and epileptic seizures, with a global incidence of 1 in 10,000 live female births [1]. Interestingly, some previous reports indicated an elevated risk of obesity and metabolic syndrome in individuals affected by RTT. However, the underlying mechanism to explain this potential association is not clear.

MeCP2 protein is expressed in all types of mammalian cells and is well known as a key epigenetic factor regulating global gene transcription. MeCP2 functions as a gene transcriptional repressor and reduces the expression of its target genes by binding to the promoter region and gathering the histone deacetylase complex, which folds the chromatin structure [2, 3]. Moreover, MeCP2 can play the opposite role as a gene transcriptional activator in cooperation with the transcription factor CREB1 [4]. Some female patients with the Zappella variants of RTT, who have verbal acquisition and manifest a mild phenotype in the neurodevelopmental aspect, have been reported to be more likely to have comorbidities of overweight and obesity [5, 6]. According to a previous report, 9% of female individuals with RTT were diagnosed as overweight [7]. Although the *MECP2* gene mutation in males can cause lethal encephalopathy, some males can survive and develop a Prader-Willi syndrome-like and autistic-like phenotype; moreover, these individuals also develop severe obesity [8, 9].

This potential association between RTT and obesity has also been demonstrated in animal experiments. In rodent models, male *Mecp2*-null mice fed a normal chow diet (ND) showed increased body weight and fat composition, along with leptin resistance without hyperphagia, whereas female heterozygous *Mecp2*-null (*Mecp2*^*+/-*^) mice and rats fed the ND gradually became overweight without hyperphagia [10–14]. Forced *Mecp2* re-expression prevented the increased body weight in male *Mecp2*-null mice, demonstrating that MeCP2 is an essential factor in regulating body weight [15]. *Mecp2* deletion induced the dysregulation of lipid metabolism along with significantly increased lipogenic enzyme gene expression, and further altered the expression of hypothalamic genes related to feeding regulation [16–20]. Collectively, these studies indicate that the *Mecp2* gene might be crucial for the epigenetic regulation of metabolic homeostasis and eating behavior in male *Mecp2*-null mice. However, the mechanism of obesity in female *Mecp2*^+/-^ mice, which were generated as an animal model of RTT, has not yet been systematically investigated.

In this study, we examined the energy metabolism, including thermogenesis in the brown adipose tissue, central feeding regulation in the hypothalamus, and dopamine reward circuitry, in female *Mecp2*^+/-^ mice. Since male *Mecp2*-null mice have a short lifespan of only about 8–10 weeks, they were not used in this study. In addition, we induced *Mecp2*^+/-^ mice to be prone to obesity by feeding a high-fat diet (HFD). We provide the first demonstration of the changes in the expression of genes and pathways related to the development of hyperphagia and extreme obesity in *Mecp2*^+/-^ mice, and further indicate a tendency of HFD palatability and excessive feeding. Collectively, these findings can provide insight into the link between RTT and obesity, and suggest targets for prevention of metabolic disease and other obesity-related complications in RTT.

## Materials and methods

### Animals and diets

The experiments were carried out using the B6.129P2(C)-*Mecp2*^*tm1*.*1Bird*^ mouse model of RTT, which was established as previously described [21]. Four-week-old female heterozygous *Mecp2*-null mice (*Mecp2*^*+/-*^ mice) and female wild-type mice as controls were purchased from Jackson Laboratory (Bar Harbor, ME, USA) and maintained on a C57BL/6J background. The mice were genotyped using polymerase chain reaction (PCR) according to a previous report [22]. Two types of diets were prepared: the ND (CLEA Rodent diet CE-2; fat: 12%, carbohydrates: 59.1%, protein: 28.8%) and the HFD (CLEA High fat diet 32; fat: 56.7%, carbohydrates: 23.1%, protein: 20%). The mice were divided into the following four groups: wild-type mice fed the ND (WT-ND mice), *Mecp2*^*+/-*^ mice fed the ND (*Mecp2*^*+/-*^-ND mice), wild-type mice fed the HFD (WT-HFD mice), and *Mecp2*^*+/-*^ mice fed the HFD (*Mecp2*^*+/-*^-HFD mice). The mice were maintained in a temperature-controlled (23°C) room on a 12-h light/dark cycle, with free access to food and water. Body weight and individual food intake were monitored twice a week. At 16 weeks, the mice were fasted overnight and anesthetized with sodium pentobarbital (50 mg/kg of intraperitoneal injection), and then blood was obtained by cardiopuncture. Plasma was separated by centrifugation at 4°C and stored at −80° until assayed. All animal experiments and care procedures were approved by the Animal Care and Use Committee of Kyoto Prefectural University of Medicine (approval number: M26-242).

### Plasma parameters

Blood glucose levels were monitored using a compact glucose analyzer (Antisense II, Horiba, Kyoto, Japan). Plasma total cholesterol (T-cho) and triglyceride (TG) levels were measured using reagents purchased from Wako (Osaka, Japan). The levels of plasma insulin and leptin were determined using enzyme-linked immunoassay kits (Morinaga Institute of Biological Science, Kanagawa, Japan; Cat. No. M1104, M1305).

### Glucose tolerance test

The mice (16 weeks old) were fasted for 16 h, and then administered an intraperitoneal glucose injection (2.0 g/kg body weight). Blood samples were collected from the tail vein at 0, 30, 60, and 120 min after the glucose injection, and the levels of blood glucose and plasma insulin were monitored as described above.

### Intraperitoneal insulin tolerance test

Mice (16 weeks old) were fasted for 2 h, and then injected intraperitoneally with insulin (0.75 IU/kg body weight) using Humulin R (Eli Lilly Japan, Kobe, Japan). The levels of blood glucose were monitored at 0, 20, 40, 60, 80, and 120 min after insulin administration as described above.

### Oxygen consumption

Oxygen consumption (VO_2_) was monitored using an O_2_/CO_2_ metabolism-measuring system (model MK-5000, Muromachi Kikai, Tokyo, Japan). This system comprised two independent 560-mL chambers for measuring two mice simultaneously, a suction pump, and a computer for data analysis. The mice were placed in the chambers, which were maintained at 23°C, and allowed to acclimate to the environment for more than two hours. The VO_2_ level was calculated as [Oa-Oc] v·m^−1^·t^−1^, where Oa is the atmospheric oxygen concentration (%) that flows into the chamber, Oc is the oxygen concentration in the chamber (%), v is the flow rate (650 mL/min), m is the mass of the mouse (kg), and t is time (h) [23, 24]. The body fat mass was determined, and oxygen consumption was then calculated by ANCOVA after adjusting for lean mass [25].

### Measurement of locomotor activity

The locomotor activity of each mouse was examined using the Supermex system (Muromachi Kikai) according to a previously described method [26]. The mice were placed in cages, maintained at 23°C, and allowed to acclimate to the environment for 24 hours. Movements of the 16-week-old mice were measured by the method of sensing the infrared radiation emitted by mice.

### Conditioned place preference (CPP) test

The preferences for the HFD in *Mecp2*^*+/-*^ mice were evaluated using the standard CPP test according to previously described methods [27]. In brief, we used two custom-made test chambers of equal size (15 × 15 × 15 cm). The walls of the light and dark chambers were white and black, lined with irregular mesh and smooth sheet floors, respectively. The pre-test was performed on Day 1 and the test was conducted on Day 6. On Days 1 and 6, each mouse was placed in the chamber with no food, and the mice were free to move through both sides for 15 min; the amount of time spent in each chamber was then measured. On Days 2–5, each mouse was conditioned individually with the HFD or ND on the light or dark side for 30 min. The preference score for the HFD was calculated as the change in the time spent in the HFD-paired chamber before and after the conditioning.

### Histology

Sections of the subcutaneous white adipose tissue (sWAT), perigonadal white adipose tissue (pWAT), interscapular brown adipose tissue (iBAT), and liver were fixed in 10% buffered formalin, and stained by hematoxylin and eosin. All images were captured by All-In-One Fluorescence Microscope BZ-X710 (Keyence, Osaka, Japan). Mean cell size and cell distribution of the sWAT and pWAT were determined from 1600 adipocytes collected from each mouse. The hepatic lipid droplets as the blank staining area in the whole area of liver section were counted using BZ Analyzer software (Keyence, Osaka, Japan).

### Quantitative reverse transcription (qRT)-PCR

Total RNA from the iBAT, hypothalamus, ventral tegmental area (VTA), and nucleus accumbens (NAc) was prepared from each sample using a Nucleospin RNA II kit (Macherey-Nagel, Düren, Germany). Template cDNA was synthesized from 500 ng of total RNA with random hexamer primers using ReverTra Ace qPCR RT Master Mix (Toyobo, Osaka, Japan). qRT-PCR was performed using SYBR Premix Ex Taq II (TaKaRa, Shiga, Japan), with 10 μM of each primer (S1 Table) in an AB 7500 Real-Time PCR System (Applied Biosystems, Tokyo, Japan). Amplification reactions were performed using the following protocol: initial activation step for 30 s at 95°C, followed by 40 cycles of 5 s at 95°C and 31 s at 60°C (delta-delta Ct method).

### Western blot analysis

Protein in the hypothalamus specimens was extracted using a radioimmunoprecipitation assay lysis buffer (Nacalai Tesque, Kyoto, Japan), and the protein concentration was determined with a Protein Quantification Assay kit (Macherey-Nagel, Düren, Germany). Tissue proteins were resolved on 10% or 12% polyacrylamide gels in the presence of sodium dodecyl sulfate, transferred electrophoretically to polyvinylidene difluoride membranes, and blocked by Blocking One reagent (Nacalai Tesque, Kyoto, Japan). The primary and secondary antibodies were diluted with Can-Get Signal (NKB-101, Toyobo). The primary antibodies were directed against phosphorylated -AMP-activated protein kinase-alpha (*p*-AMPKα; 1:5000; #2535, Cell Signaling Technology Japan, Tokyo, Japan), AMPKα (1:5000, #5831, Cell Signaling Technology Japan, Tokyo, Japan), phosphorylated-forkhead-box protein O1 (*p*-FoxO1; 1:5000, #9461, Cell Signaling Technology Japan), FoxO1 (1:5000; #9454, Cell Signaling Technology Japan), phosphorylated-signal transducer and activator of transcription 3 (*p*-STAT3; 1:5000, #9131, Cell Signaling Technology Japan), STAT3 (1:5000; #9132, Cell signaling Technology Japan), MeCP2 (1:5000; #3456, Cell Signaling Technology Japan), Agouti-related peptide (AgRP; 1:3000, #AF634, R&D Systems, Minneapolis, MN, USA), proopiomelanocortin (POMC; 1:3000, #ab32893, Abcam, Cambridge, UK), and β actin (1:15,000; #3700, Cell Signaling Technology Japan). Immunoblot signals were confirmed using ImageQuant LAS 500 Gel Documentation System (GE Healthcare UK Ltd., Buckinghamshire, UK).

### Statistical analysis

All data are shown as the mean ± standard error of the mean. Parameters were analyzed by one-way ANOVA or two-way ANOVA or ANCOVA. Two-way ANOVA was used to compare the effect of genotypes (WT and *Mecp2*^+/-^) and diets (ND and HFD). When a significant interaction was obtained, *post-hoc* analysis was conducted with a Turkey–Kramer multiple comparison test. Regarding the CPP test, we used the Student’s *t*-test for comparisons between the two groups. The statistical details (p-value, F, and degree of freedom (Df)) are based on the results of the two-way ANOVA testing. P < 0.05 was considered to indicate a statistically significant difference.

## Results

### *Mecp2*^*+/-*^ mice fed the HFD showed significant obesity with hyperphagia

**Fig 1:**
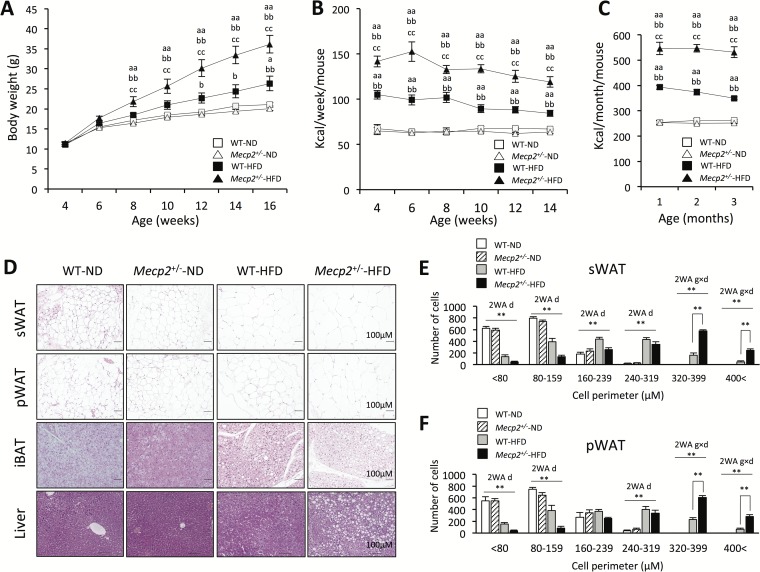
Body weight gain, food intake, and adiposity in *Mecp2*^+/-^ mice fed a high-fat diet. (A) Body weight change. (B) Weekly food intake. (C) Monthly food intake. Values are the mean ± SEM for 9–11 mice. (D) Histology of the subcutaneous white adipose tissue (sWAT), perigonadal white adipose tissue (pWAT), brown adipose tissue, and liver. (E) Distribution of adipocyte perimeters in the sWAT. (F) Distribution of adipocyte perimeters in the pWAT. (A–F) Mice were tested at 16 weeks of age; values are the mean ± SEM for 7–8 mice. * = p < 0.05, ** = p < 0.01, ^a^p < 0.05, ^aa^p < 0.01 vs. WT mice fed a normal chow diet (WT-ND). ^b^p < 0.05, ^bb^p < 0.01 vs. *Mecp2*^+/-^ mice fed a normal chow diet (*Mecp2*^+/-^-ND). ^c^p < 0.05, ^cc^p < 0.01 vs. WT mice fed a high-fat diet (WT-HFD). 2WA = Two-way ANOVA, g = genotype, d = diet, Two-way ANOVA followed by *post-hoc* Tukey-Kramer Multiple-Comparison test.

The initial body weight from 4 to 16 weeks of age did not differ between the WT-ND and *Mecp2*^*+/-*^-ND mice. However, the *Mecp2*^*+/-*^-HFD mice showed overt obesity compared with WT-HFD mice from 8 to 16 weeks of age (p < 0.01). As of 16 weeks, the body weight of WT-HFD mice was significantly higher than that of WT-ND mice (p < 0.05). There was a significant interaction seen by measuring the body weight between genotype and diet (interaction: p < 0.01, F = 5.11, Df = 28; Fig 1A). Moreover, *Mecp2*^*+/-*^-HFD mice showed significantly increased food intake compared with WT-HFD mice from the age of weaning (p < 0.01), whereas no difference was observed in food intake between WT-ND and *Mecp2*^*+/-*^-ND mice. There was a significant interaction in the food intake between genotype and diet (interaction: p < 0.01; F = 28.4; Df = 31) (Fig 1B and 1C, S1A Fig). With regard to body composition, the weights of the sWAT and pWAT did not differ between *Mecp2*^*+/-*^-ND and WT-ND mice, whereas the weights of both the sWAT and pWAT were significantly greater in *Mecp2*^*+/-*^-HFD mice compared with those in WT-HFD mice (p < 0.01) (Table 1). There were significant interaction in the weights of both the sWAT and pWAT between genotype and diet (interactions: p < 0.05; F = 19.80; Df = 28, and p < 0.05; F = 4.93; Df = 28). In the histological distribution patterns, mean perimeter of adipocytes in the sWAT and pWAT were not different between *Mecp2*^*+/-*^-ND and WT-ND mice, whereas mean perimeter of adipocytes in the sWAT and pWAT were larger in *Mecp2*^*+/-*^-HFD mice compared with those in WT-HFD mice. There was a significant interaction in the perimeter of adipocytes more than 320 μM in the sWAT and pWAT between genotype and diet (interactions: p < 0.01; F = 89.4; Df = 24, and p < 0.01; F = 60.2; Df = 24; Fig 1D–1F). Histology of the liver showed that *Mecp2*^*+/-*^-HFD mice had a more severe fatty liver, similar to that of non-alcoholic fatty liver disease, compared with WT-HFD mice, whereas there were no differences in the livers of *Mecp2*^*+/-*^-ND and WT-ND mice. There was a significant interaction in the blank staining of histology of the liver between genotype and diet (interaction: p < 0.05; F = 4.91; Df = 12; Fig 1D, S1B Fig). Overall, these results indicated that *Mecp2*^*+/-*^ mice developed extreme obesity with hyperphagia and a significantly increased fat composition under the HFD condition.

**Table 1:** Organ weights and metabolic parameters.

	n	WT-ND	*Mecp2*^*+/-*^-ND	WT-HFD	*Mecp2*^*+/-*^-HFD
Body Weight (g)	8	20.3±0.5	19±0.7	26.1±2.6 ^a^	34.7±2.8 ^aa^ ^,^^bb^^,^ ^cc^
sWAT mass (g)	8	0.20±0.04	0.57±0.15	0.95±0.19 ^a^	2.03±0.23 ^aa^ ^,^^bb^^,^ ^cc^
pWAT mass (g)	8	0.12±0.03	0.46±0.13	0.85±0.2 ^a^	1.94±0.30 ^aa^^,^ ^bb^^,^ ^cc^
BAT mass (g)	8	0.067±0.004	0.061±0.004	0.109±0.013	0.089±0.012
Liver (g)	8	0.68±0.05	0.81±0.04	1.01±0.13	1.53±0.28 ^aa^^,^ ^b^
Spleen (g)	8	0.07±0.01	0.06±0.01	0.10±0.01	0.08±0.01
Brain (g)	8	0.37±0.02	0.34±0.01	0.38±0.02	0.35±0.01
sWAT/BW (%)	8	0.76±0.17	1.85±0.49	1.79±0.45	3.92±0.45 ^aa^ ^,^^bb^^,^ ^cc^
pWAT/BW (%)	8	0.57±0.11	2.23±0.60	2.28±0.59	4.77±0.66 ^aa^ ^,^^b^^,^ ^c^
BAT/BW (%)	8	0.34±0.03	0.31±0.02	0.35±0.04	0.30±0.03
Liver/BW (%)	8	3.39±0.29	4.12±0.21	3.84±0.24	4.22±0.41
Spleen/BW (%)	8	0.35±0.02	0.29±0.03	0.35±0.03	0.22±0.02
Brain/BW (%)	8	1.83±0.10	1.74±0.10	1.52±0.12	1.04±0.08
T-cho (mg/dl)	8	57.7±3.9	59.9±5.1	89.3±11.1	144.3±16.4 ^aa^^,^ ^bb^^,^ ^cc^
TG (mg/dl)	8	13±2.3	14.3±1.8	17±2.3	19.1±4.9
Glucose (mg/dl)	6–7	71±7	79±14	130±10 ^aa^^,^ ^b^	192±10 ^aa^^,^ ^bb^^,^ ^c^
Insulin (μIU/ml)	6–7	8.4±1.0	11.5±0.9	16.1±2.1	25.7±3.1
HOMA-IR	6–7	1.5±0.2	2.2±0.4	5.3±0.9 ^a^^,^ ^b^	11.9±1.4 ^aa^^,^ ^bb^^,^ ^cc^
Leptin (ng/ml)	6	1.5±0.3	7.3±0.7 ^a^	12.9±2.3 ^aa^^,^ ^b^	25.1±1.3 ^aa^^,^ ^bb^^,^ ^cc^

The values are the mean±SE. Two-way ANOVA followed by *post-hoc* Tukey-Kramer Multiple-Comparison test.

^a^p < 0.05

^aa^p < 0.01, vs. WT mice fed the normal chow diet (WT-ND).

^b^p < 0.05

^bb^p < 0.01, vs. *Mecp2*^*+/-*^ mice fed normal chow diet (*Mecp2*^*+/-*^-ND).

^c^p < 0.05

^cc^p < 0.01, vs. WT mice fed the high fat diet (WT-HFD).

### *Mecp2*^*+/-*^ mice fed the HFD showed a remarkable increase in plasma leptin levels, glucose intolerance, and insulin resistance

**Fig 2:**
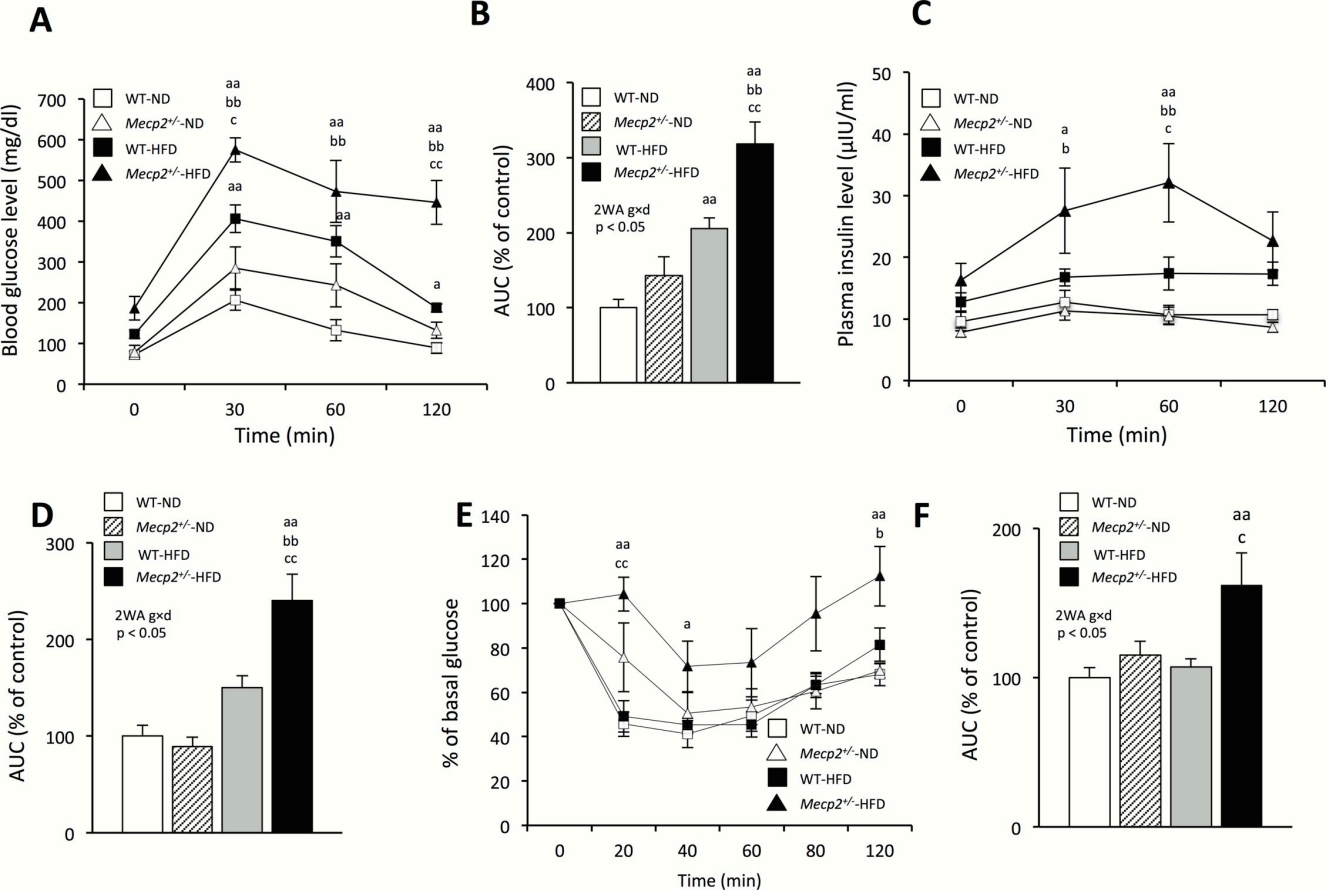
Glucose intolerance and insulin resistance in *Mecp2*^+/-^ mice. (A) Intra-peritoneal glucose tolerance tests (IPGTTs) to determine blood glucose levels. (B) Quantification of the area under the curve (AUC) in (A). (C) IPGTTs for determining plasma insulin levels. (D) Quantification of the AUC in (C). (E) Insulin tolerance tests (ITTs). (F) Quantification of the AUC in (E). Mice were tested at 16 weeks of age; values are the mean ± SEM for 6–7 mice. ^a^p < 0.05, ^aa^p < 0.01 vs. WT mice fed a normal chow diet (WT-ND). ^b^p < 0.05, ^bb^p < 0.01 vs. *Mecp2*^+/-^ mice fed a normal chow diet (*Mecp2*^+/-^-ND). ^c^p < 0.05, ^cc^p < 0.01 vs. WT mice fed a high-fat diet (WT-HFD). 2WA = Two-way ANOVA, g = genotype, d = diet, Two-way ANOVA followed by *post-hoc* Tukey-Kramer Multiple-Comparison test.

We next examined the effects of MeCP2 deficiency and the HFD condition on metabolic parameters. There were significant interactions in the blood glucose (interaction: p < 0.05; F = 6.04; Df = 21), homeostatic model assessment-insulin resistance (HOMA-IR) (interaction: p < 0.01; F = 10.54; Df = 21), T-cho (interaction: p < 0.01; F = 8.10; Df = 30) and leptin levels (interaction: p < 0.05; F = 5.37; Df = 20) between genotype and diet. There were no significant interactions in the plasma TG and insulin levels between genotype and diet. However, plasma insulin levels were significantly increased by genotype (p < 0.01; F = 10.72; Df = 21) and HFD feeding (p < 0.01; F = 27.78; Df = 21) independently of each other. Blood glucose, HOMA-IR, T-cho levels did not differ between *Mecp2*^*+/-*^-ND and WT-ND mice. However, the plasma leptin levels of *Mecp2*^*+/-*^-ND mice were significantly higher than those of WT-ND mice (p < 0.05), and the blood glucose levels, HOMA-IR, and plasma leptin levels of WT-HFD mice were significantly higher than those of WT-ND mice (p < 0.01). Moreover, the blood glucose, HOMA-IR, T-cho, and plasma leptin levels of *Mecp2*^*+/-*^-HFD mice were significantly higher than those of *Mecp2*^*+/-*^-ND mice (p < 0.01). The blood glucose levels, HOMA-IR, plasma leptin, and T-cho levels of *Mecp2*^*+/-*^-HFD mice were significantly higher than those of WT-HFD mice (p < 0.05; Table 1). Intraperitoneal glucose tolerance testing and insulin tolerance testing demonstrated the greatest impairment of blood glucose and serum insulin levels in *Mecp2*^*+/-*^-HFD mice among the four groups. There were significant interactions in the AUC of IPGTTs for determined blood glucose levels and plasma insulin levels and AUC of ITTs (interactions: p < 0.05; F = 4.68; Df = 20, p < 0.05; F = 7.91; Df = 20, and p < 0.05; F = 5.01; Df = 22). Overall, *Mecp2*^*+/-*^-HFD mice showed notable hyperleptinemia, lower glucose tolerance, and higher insulin tolerance than WT-HFD mice (Fig 2A–2F).

### MeCP2 does not affect energy expenditure and locomotor activity in HFD-fed mice

**Fig 3:**
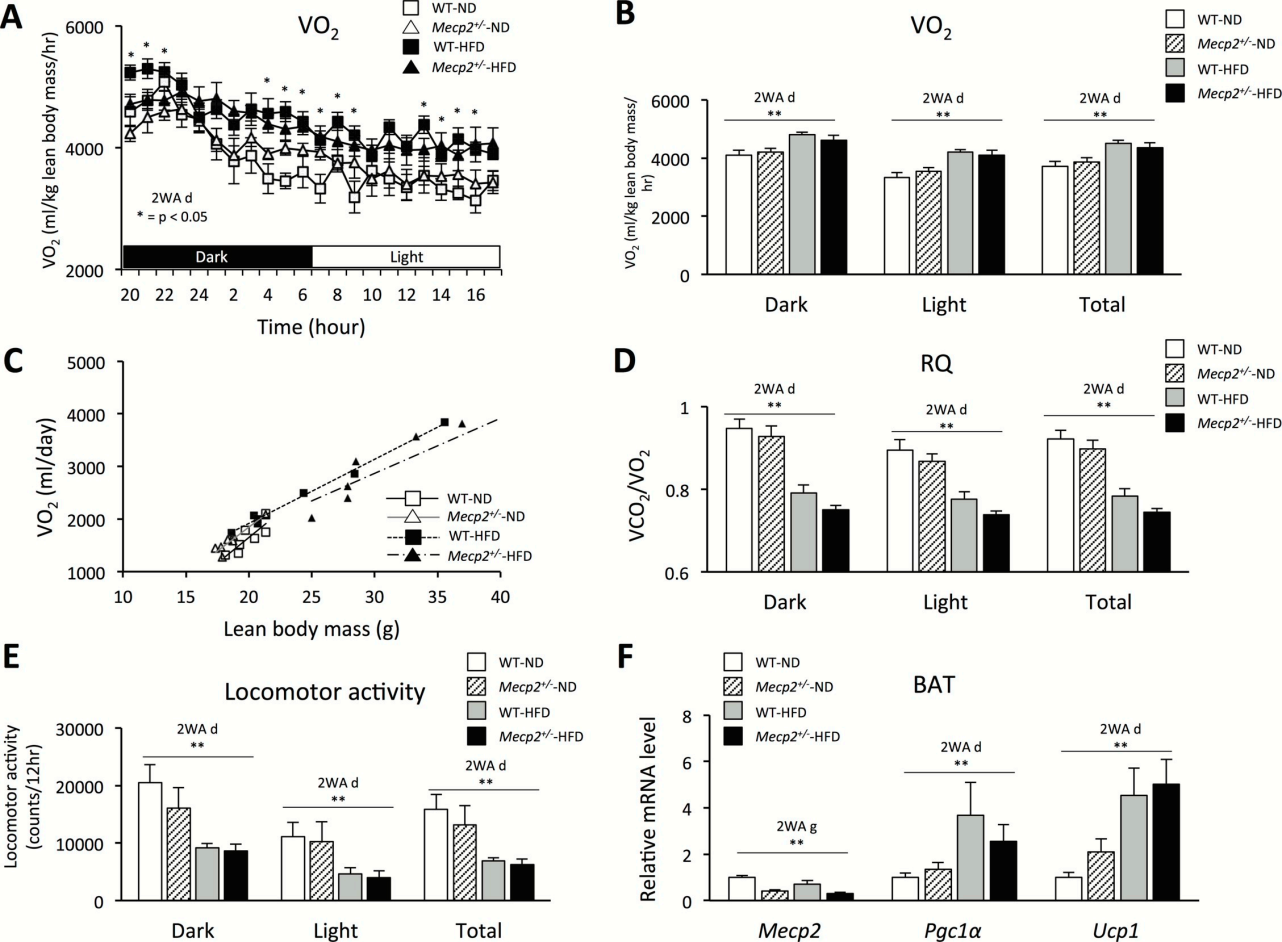
Oxygen consumption, locomotor activity, and brown adipose tissue-related gene expression in *Mecp2*^+/-^ mice. (A) Twenty-two-hour oxygen consumption adjusted by lean body mass. (B) Oxygen consumption in dark and light phase. (C) Oxygen consumption analyzed by ANCOVA. (D) Respiratory quotient (VCO_2_/VO_2_). (E) Locomotor activity. (F) *Mecp*2, *Ucp1 and Pgc1α* mRNA expression in the BAT using qPCR. (A–F) Mice were tested at 16 weeks of age; values are the mean ± SEM for 7–8 mice. * = p < 0.05, ^a^p < 0.05, ** = p < 0.01, 2WA = Two-way ANOVA, g = genotype, d = diet, Two-way ANOVA followed by *post-hoc* Tukey-Kramer Multiple-Comparison test.

Oxygen consumption, an indirect measurement of metabolism, respiratory quotient (RQ) and locomotor activity, did not differ between *Mecp2*^*+/-*^ and WT mice in both the dark and light stages in each dietary condition. Oxygen consumption was adjusted to lean body mass (kg) and calculated by ANCOVA (Fig 3A–3C). There was no significant interaction between genotype and diet in terms of oxygen consumption, RQ and locomotor activity (Fig 3A–3E). However, significant effects of diet, independent of genotype, were found on oxygen consumption in the dark, light and total stages (p < 0.01; F = 14.68; Df = 24, p < 0.01; F = 15.89; Df = 24, and p < 0.01; F = 17.63; Df = 24; Fig 3B), Moreover, significant effects of diet, independent of genotype, were found on RQ in the dark, light and total stages (p < 0.01; F = 70.23; Df = 24, p < 0.01; F = 43.20; Df = 24, and p < 0.01; F = 62.78; Df = 24; Fig 3D). Significant effects of diet, independent of genotype, were found in the locomotor activity in the dark, light and total stages (p < 0.01; F = 14.82; Df = 24, p < 0.01; F = 13.14; Df = 24, and p < 0.01; F = 12.88; Df = 24; Fig 3E). We quantitatively examined the *Mecp2* mRNA expression in the iBAT, Significant effects of genotype, independent of diet, were found on *Mecp2* mRNA expression levels (p < 0.01; F = 21.03; Df = 20; Fig 3F). Significant effects of diet, independent of genotype, were found in the mRNA expression levels of uncoupling protein 1 (*Ucp1*), an essential protein for BAT thermogenesis, and peroxisome proliferator-activated receptor gamma coactivator 1-alpha (*Pgc1α*), a transcriptional co-activator for *Ucp1* expression (p < 0.01; F = 9.69; Df = 20, and p < 0.01; F = 11.4; Df = 22; Fig 3F). Similarly, the histological findings of the iBAT were similar between *Mecp2*^+/-^ and WT mice in each dietary condition (Fig 1D).

### *Mecp2*^*+/-*^ mice fed the HFD showed disturbance in hypothalamic regulation of food intake

**Fig 4:**
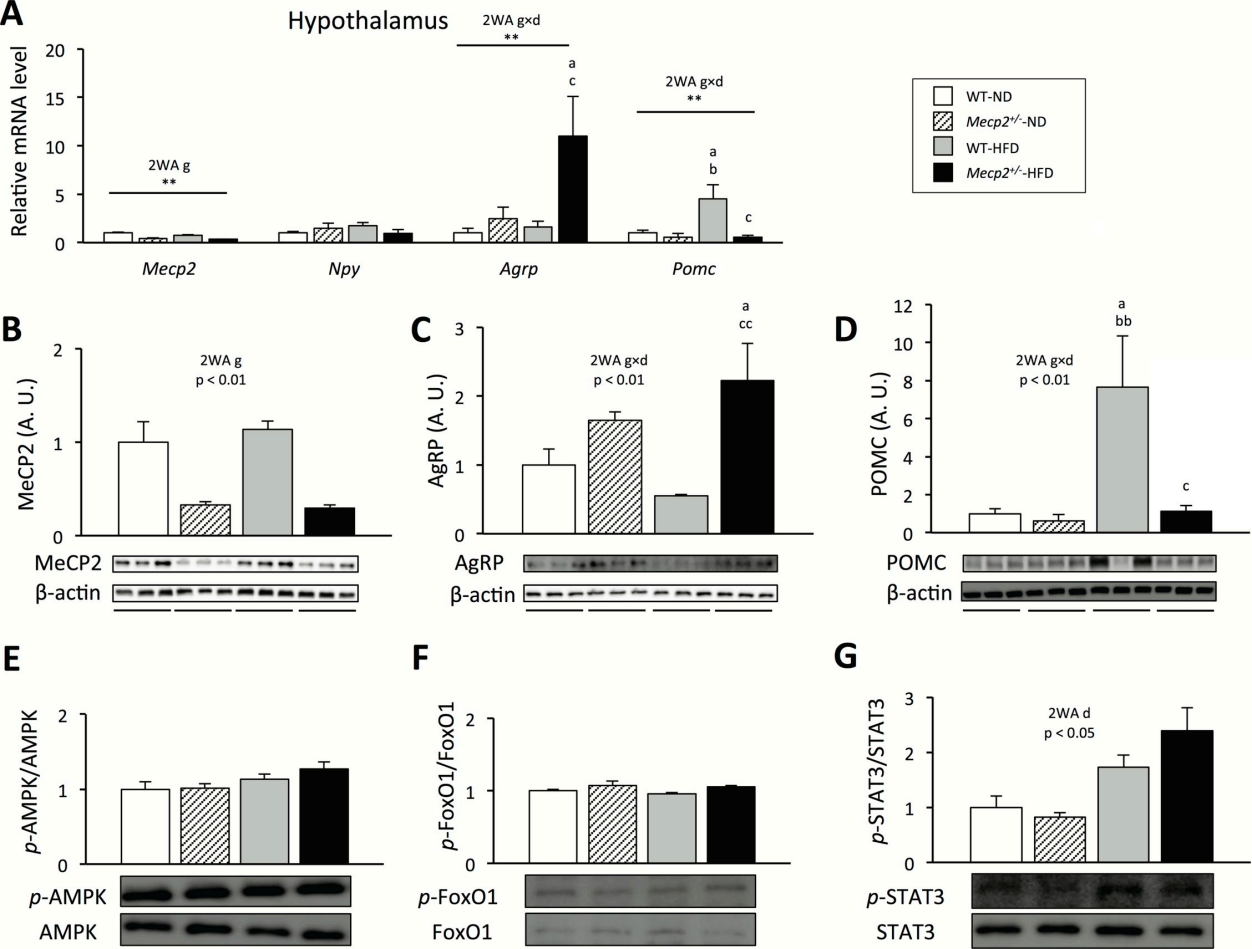
Hypothalamic dysregulation of food intake in *Mecp2*^+/-^ mice. (A) mRNA expression levels of food intake regulation-related genes in the hypothalamus, determined using qPCR. (B) MeCP2, (C) AgRP, and (D) POMC protein expression in the hypothalamus, determined using western blot analysis. Phosphorylation of (E) AMPK, (F) FoxO1, and (G) STAT3 in the hypothalamus, using western blot analysis. (A–F) Mice were tested at 16 weeks of age; values are the mean ± SE for 5 mice. ** = p < 0.01, ^a^p < 0.05, vs. WT mice fed a normal chow diet (WT-ND). ^b^p < 0.05, ^bb^p < 0.01 vs. *Mecp2*^*+/-*^ mice fed a normal chow diet (*Mecp2*^*+/-*^-ND). ^c^p < 0.05, ^cc^p < 0.01 vs. WT mice fed a high-fat diet (WT-HFD). 2WA = Two-way ANOVA, g = genotype, d = diet, Two-way ANOVA followed by *post-hoc* Tukey-Kramer Multiple-Comparison test.

To further determine the cause of the hyperphagia in *Mecp2*^*+/-*^-HFD mice, we examined the expression of hypothalamic neuropeptides that are known to regulate homeostatic feeding behavior in *Mecp2*^+/-^ mice. *Mecp2* mRNA and protein expression levels in the hypothalamus were significantly decreased in *Mecp2*^*+/-*^ compared to WT mice in each dietary condition. A significant effect of genotype independent diet was found in *Mecp2* mRNA expression levels in the hypothalamus (p < 0.01; F = 34.72; Df = 24; Fig 4A). Moreover, a significant effect of genotype independent diet was found in *Mecp2* protein expression levels in the hypothalamus (p < 0.01; F = 53.83; Df = 20; Fig 4B). Phosphorylation of AMPK/FoxO1 and STAT3 protein; levels of neuropeptide Y (*Npy*), *Agrp*, and *Pomc* mRNAs; and levels of AgRP and POMC proteins in the hypothalamus did not differ between *Mecp2*^*+/-*^-ND and WT-ND mice. However, under the HFD condition, the *Agrp* mRNA expression level was significantly higher (p < 0.05) and the *Pomc* mRNA expression level was significantly lower (p < 0.05) in the hypothalamus of *Mecp2*^*+/-*^ mice compared with those in WT mice (Fig 4A–4G). There were significant interactions in the *Agrp* and *Pomc* mRNA expression levels in the hypothalamus between genotype and diet (interactions: p < 0.05; F = 5.23; Df = 16, and p < 0.05; F = 5.51; Df = 16; Fig 4A). The phosphorylation levels of AMPK/FoxO1 did not differ between *Mecp2*^*+/-*^-HFD and WT-HFD mice, whereas the phosphorylation of STAT3 tended to be higher (p = 0.13). A significant effect of diet independent of genotype was found in the phosphorylation of STAT3 (p < 0.01; F = 2.71; Df = 16; Fig 4E–4G). AgRP protein expression was significantly higher (p < 0.01), and POMC protein expression was significantly lower (p < 0.01) in *Mecp2*^*+/-*^-HFD mice than in WT-HFD mice. There were significant interactions in the *Agrp* and *Pomc* protein expression levels in the hypothalamus between genotype and diet (interactions: p < 0.05; F = 4.56; Df = 16, and p < 0.05; F = 5.15; Df = 16; Fig 4C and 4D).

### The mesolimbic dopamine circuit is disturbed in *Mecp2*^*+/-*^ mice fed the HFD

**Fig 5:**
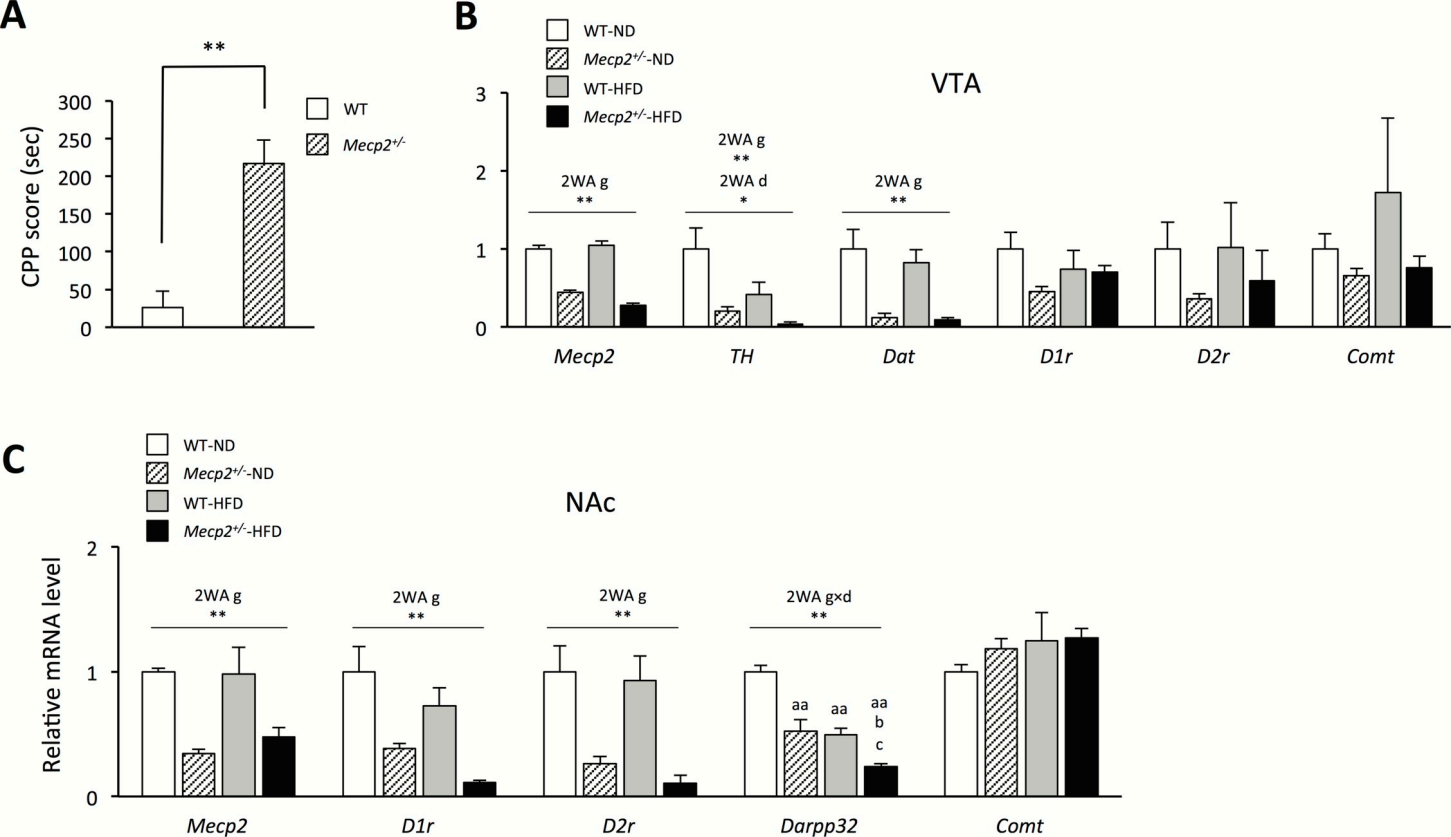
Dysregulation of the mesolimbic dopamine circuitry in *Mecp2*^+/-^ mice. (A) Conditioned place preference (CPP) test; mice were tested at 10–12 weeks of age. Values are the mean ± SEM for 8 mice. **p < 0.01 vs. WT mice. (B) *Mecp2* and dopamine-related gene expression in the VTA, determined using qPCR. (C) Dopamine-related gene expression in the NAc, determined using qPCR. (B–C) Mice were tested at 16 weeks of age; values are the mean ± SEM for five mice. * = p < 0.05, ^a^p < 0.05, ** = p < 0.01, ^aa^p < 0.01 vs. WT mice fed a normal chow diet (WT-ND). ^b^p < 0.05, vs. *Mecp2*^+/-^ mice fed a normal chow diet (*Mecp2*^+/-^-ND). ^c^p < 0.05, vs. WT mice fed a high-fat diet (WT-HFD). 2WA = Two-way ANOVA, g = genotype, d = diet, Two-way ANOVA followed by *post-hoc* Tukey-Kramer Multiple-Comparison test.

Given the essential role of the mesolimbic dopamine circuit in hedonic feeding behavior and regulation of the palatability of the HFD, we also investigated the expression of dopaminergic reward circuitry-related genes in *Mecp2*^+/-^ mice. The CPP test revealed that *Mecp2*^*+/-*^-ND mice greatly preferred the HFD (p < 0.01; Fig 5A). *Mecp2* mRNA expression levels in the VTA and NAc were significantly decreased in *Mecp2*^*+/-*^ compared to WT mice, regardless of the dietary condition. A significant effect of genotype independent diet was found in the *Mecp2* mRNA expression levels in the VTA and NAc (p < 0.01; F = 6.67; Df = 16; Fig 5B, p < 0.01; F = 23.57; Df = 16; Fig 5C). In the VTA, significant effect of genotype independent diet were found in the mRNA expression levels of tyrosine hydroxylase (*TH*) and dopamine transporter (*Dat*) (p < 0.01; F = 33.05; Df = 16, and p < 0.01; F = 48.49; Df = 16). Moreover, significant effect of diet independent genotype were found in the mRNA expression levels of *TH* (p < 0.05; F = 6.81; Df = 16). However, the mRNA expression level of the catechol-*O*-methyltransferase gene (*Comt*), which encodes the main enzyme that degrades dopamine, dopamine receptor D1 (*D1r*) and dopamine receptor D2 (*D2r*) did not differ among the groups (Fig 5B). In the NAc, a significant effect of genotype independent diet was found in *D1r* and *D2r* mRNA expression (p < 0.01; F = 27.93; Df = 16, p < 0.01; p < 0.01; F = 27.61; Df = 16). Thus, *D1r* and *D2r* mRNA expression in the NAc was significantly lower in *Mecp2*^*+/-*^ mice than in WT mice in each dietary condition. Interestingly, there was a significant interaction in the expression levels of dopamine- and cAMP-regulated phosphoprotein 32 (*Darpp32*), which is expressed downstream of D1R and D2R signaling and has a pivotal role in dopaminergic signal transmission, in the NAc between genotype and diet (interaction: p < 0.05; F = 5.78; Df = 16). *Darpp32* mRNA expression level in the NAc was significantly lower in *Mecp2*^*+/-*^-ND mice than in WT-ND mice (p < 0.01), and significantly lower in *Mecp2*^*+/-*^-HFD mice than in *Mecp2*^*+/-*^-ND mice (p < 0.05). Within the HFD condition, the *Darpp32* mRNA expression level was significantly lower in *Mecp2*^*+/-*^ mice than in WT mice (p < 0.05), and there was no difference in *Comt* mRNA expression levels in the NAc (Fig 5C). Overall, these results indicated that mesolimbic dopamine-related gene expression decreased in *Mecp2*^*+/-*^ mice, and the HFD condition enhanced this downregulation in the reward circuitry. *Mecp2*^*+/-*^ mice exhibited a reward effect for the HFD.

## Discussion

We here provide the first demonstration that *Mecp2*^+/-^-HFD mice display prominent extreme obesity with hyperphagia and show a strong preference for the HFD compared with WT-HFD mice. In addition, *Mecp2*^+/-^-HFD mice showed more severe subcutaneous and visceral fat composition, fatty liver, glucose intolerance, insulin resistance, and leptin resistance than WT-HFD mice. Based on these results, we propose that an increase in calorie intake under the HFD condition was the inducible cause of the extreme obesity in *Mecp2*^+/-^ mice since there was no difference in body weight and calorie intake between *Mecp2*^+/-^-ND and WT-ND mice. A previous study showed that an *Mecp2*-null rodent model fed an ND showed increased body fat composition and energy metabolic dysfunction, including lipid metabolism deficiency [10, 12, 14, 16]. However, our results suggest that disturbance of homeostatic feeding regulation in the hypothalamus and hedonic feeding regulation in the reward circuitry are largely responsible for the obesity in *Mecp2*^+/-^-HFD mice.

Resting energy expenditure and locomotor activity are important factors in the development of obesity. Resting energy expenditure is actively controlled by the sympathetic nervous system in the hypothalamus [28], and VO_2_ is an important index for resting energy expenditure with BAT thermogenesis. The expression of UCP1 and PGC1α, which play pivotal roles in thermogenesis, is reportedly activated by β-adrenaline receptor signaling in brown adipose cells via hypothalamic sympathetic nervous activity [29]. Previous studies demonstrated that daily oxygen consumption and locomotor activity decreased in male *Mecp2*-null mice [16, 30]. However, we did not find a difference in oxygen consumption or locomotor activity between WT mice and *Mecp2*^+/-^ mice. Moreover, the expression levels of *Ucp1* and *Pgc1α* mRNA in the BAT did not differ between WT mice and *Mecp2*^+/-^ mice. Based on these results, we consider that the production of thermogenic protein was not impaired in *Mecp2*^+/-^ mice. Thus, our results suggested that MeCP2 deficiency does not appear to influence energy expenditure or physical activity in the *Mecp2*^+/-^ mice, even when fed an HFD.

In the hypothalamus, the phosphorylation of AMPK/FoxO1, located downstream of the ghrelin receptor signaling in the orexigenic pathway, promotes NPY/AgRP expression in the arcuate nucleus (ARC) [31–33]. Conversely, the phosphorylation of STAT3, located downstream of leptin receptor signaling in the anorexigenic pathway, promotes POMC expression in the ARC [34]. Thus, NPY/AgRP promotes appetite, whereas POMC promotes satiety for the control of body weight balance. Previous reports have revealed underexpression of NPY/AgRP mRNA and overexpression of POMC mRNA in the hypothalamus of HFD-fed wild-type C57BL/6J mice [35, 36]. Our data showed that the expression levels of AgRP and POMC in the hypothalamus were significantly higher and lower, respectively, in *Mecp2*^+/-^-HFD mice compared with those of WT-HFD mice, although the phosphorylation levels of AMPK/FoxO1 did not differ between the groups. However, plasma leptin level was notably higher, and phosphorylation levels of STAT3 in the hypothalamus was tended to be higher in the *Mecp2*^+/-^-HFD mice compared with that in the WT-HFD mice. Leptin is a hormone produced by white adipose cells and plays a pivotal role in the inhibition of appetite by binding to leptin receptors in the hypothalamus. A previous report revealed that male *Mecp2*-null mice fed an ND showed leptin resistance, and POMC expression in the hypothalamus could not be recovered even by intraventricular leptin administration [10]. Moreover, another study showed that MeCP2 promotes the transcription of POMC and regulates POMC expression [20]. We found that POMC expression in the hypothalamus was impaired in *Mecp2*^+/-^-HFD mice, although STAT3 phosphorylation was increased in response to the high levels of circulating leptin. This result suggests that the hyperphagia with hyperleptinemia in *Mecp2*^+/-^-HFD mice could be due to the impairment of POMC expression in *Mecp2*^+/-^-HFD mice, which mechanism might be influenced by methylation profile in the POMC promoter induced by high-fat diet condition, but not by impairment of leptin receptor/STAT3 signaling. In addition, overexpression of AgRP in the hypothalamus might also have contributed to the increased appetite of *Mecp2*^+/-^-HFD mice. The ND condition did not affect AgRP and POMC expression between *Mecp2*^+/-^ and WT mice, whereas the HFD condition induced severe dysregulation of AgRP and POMC expression in *Mecp2*^+/-^ mice.

In the reward circuitry, dopaminergic neurons project from the VTA to the NAc and play a pivotal role in the hedonic regulation of feeding behavior [37]. A relation between addiction-like eating of an HFD and reward circuitry dysfunction has been reported. For example, dopamine activity dysfunction in the VTA was shown to be remarkably induced as a result of HFD consumption [38]. In addition, the lentivirus-mediated knockdown of striatal D2R gene expression boosted the compulsive eating and palatability of a cafeteria diet [39]. Furthermore, long-term HFD consumption induced the downregulation of TH and DAT expression in the VTA, and the downregulation of dopamine D1R and D2R receptor expression in the NAc, which were associated with palatability of an HFD accompanied by decreased satisfaction with a normal amount of HFD [34, 40–42]. TH plays an essential role as a rate-limiting enzyme of dopamine synthesis, and DAT is a transmembrane protein for dopaminergic transmission responsible for the reuptake of dopamine in the VTA. Dopaminergic signaling from the VTA regulates dopamine receptor expression and dopaminergic transmission in the NAc. Our data demonstrated that *Mecp2*^+/-^-ND mice showed overall impairment of dopaminergic gene expression in the reward circuitry and a significant preference for the HFD in the CPP test. These phenomena suggest that dopamine reward circuitry dysfunction in

*Mecp2*^+/-^ mice is associated with the HFD palatability and addiction-like eating.

The role of MeCP2 in the reward circuitry has not been clearly elucidated until now. Although a deficit of MeCP2 in the reward circuitry has been shown to induce drug addiction, to our knowledge, this is the first study to investigate the relation between a deficit of MeCP2 and food addiction [43, 44]. MeCP2 protein regulates TH expression by directly binding to the promoter region of the *TH* gene, which then decreases TH expression levels of male *Mecp2*-null mice and female *Mecp2*^+/-^ mice [45, 46]. These results support the hypothesis that MeCP2 regulates the dopamine reward circuitry function via TH expression and dopamine synthesis. In our study, *Mecp2*^+/-^-ND mice showed significantly decreased *TH* and *Dat* mRNA expression levels in the VTA, along with decreased *D1r*, *D2r*, and *Darpp32* expression levels in the NAc compared with those of WT-ND mice. In other words, *Mecp2*^*+/-*^-ND mice had downregulated dopamine synthesis and reuptake in the VTA, along with downregulated dopamine receptor signaling in the NAc. We suggest that the decreased *Darpp32* expression in *Mecp2*^*+/-*^ mice enhanced the decreased D1R and D2R signaling in the NAc. Moreover, there was a significant interaction in the *Darpp32* m RNA expression between the genotype of *Mecp2*^+/-^ mice and their dietary condition. Consequently, *Mecp2*^+/-^ mice showed significantly decreased *TH* and *Dat* mRNA expression in the VTA, as well as decreased *D1r*, *D2r*, and *Darpp32* mRNA expression levels in the NAc compared with those of WT mice, resulting in a greater appetite for the HFD. These results suggest that a decrease in MeCP2 expression in an HFD condition induced very severe dysfunction of the dopamine reward circuitry.

In summary, HFD feeding induced collapse of food intake regulation in the hypothalamus and dopamine reward circuitry, and accelerated the development of extreme obesity associated with addiction-like eating behavior in *Mecp2*^*+/-*^ mice. Based on our results, we consider the hypothesis that the easy access to palatable high-fat foods in the modern lifestyle could induce early-onset obesity in humans with RTT.

## Supporting information

S1 Table Sequences of primer pairs for quantitative RT-PCR. (TIFF)

S1 Fig A, Weekly Food intake (g/week). B, Percentage of the hepatic lipid droplets area (Blank area in H&E staining).
(TIFF)
